# Heuristics in Judgment Tasks with Unrecognized Elements

**DOI:** 10.5964/ejop.v15i3.1687

**Published:** 2019-09-27

**Authors:** Miguel Dimase

**Affiliations:** a Faculty of Psychology, University of Buenos Aires, Argentina; Edinburgh Napier University, United Kingdom

**Keywords:** heuristics, recognition, bounded rationality, decision-making

## Abstract

According to published studies in the field, random choice and random estimation are the only options for tackling judgment and decision-making tasks where the elements from which to infer a required criteria are not recognized. In Campitelli and Labollita (2010), participants were asked to estimate the nationality and Elo rating of chess players based on their surnames. In the present study I re-analyze those 123 participants from Campitelli and Labollita (2010) who declared not to have recognized any player. Even in this scenario of null recognition, they managed to correctly infer the Russian players' nationality and Elo ratings; it is likely that successful and ecologically rational heuristics were used. I found evidence of new structured probabilistic environments external to the lab, likely to have generated a number of undirected and involuntary associations in the memories of the participants, who may have used them in their heuristics to infer the criteria requested. The results support the models of limited rationality: despite the scarcity of available information, the fact that the heuristics did not guarantee success, and the risk of overestimating the heuristics’ effectiveness while underestimating their own biases, participants still favored them over random guesswork, thus suggesting an adaptive use. I invite a revision of what is considered “good reasoning” when applied to problems in environments of uncertainty that call for satisfactory, rather than optimal, solutions. This research provides the basis for new studies in the field of heuristics under previously unexplored conditions, and a new perspective for the analysis of prior works, towards a better understanding of the relationship between cognition and the environment.

Historically, philosophy and psychology have tried to define human rationality in terms of its relationship with the rules of logic, offering models that ranged from complete adherence to total independence from said rules. Meanwhile, research on how people make judgments and decisions have generally relied on the criteria of coherence, correspondence and pragmatism for the purposes of gauging truthness and correctness (Dunwoody, 2009). Research so far has mostly used the criterion of coherence to evaluate the truthness/correctness or falsehood/incorrectness of a given process against a normative system. The program *Heuristics and biases* initiated by Kahneman and Tversky (e.g., Kahneman, Slovic, & Tversky, 1982; Kahneman & Tversky, 1973) applied this criterion when it concluded that humans’ heuristics (inferential strategies that guide the search for solutions) exhibit biases (systematic errors) compared to the norms of classical logic and probability. By contrast, the program *Fast-and-frugal heuristics*^1^ initiated by Gerd Gigerenzer (e.g., Gigerenzer & Todd, 1999) used the criteria of correspondence (coincidence with facts of the world) and pragmatism (utility) to characterize these heuristics as inferential abilities adapted to cognitive limitations and varying environments. The recognition heuristic (Gigerenzer & Goldstein, 1996) used these criteria for evaluating forced decisions such as the following:

Which of these German cities has the largest population?

(a) Hamburg
(b) Kassel

The heuristic was defined thus: "If one of the two objects is recognized and the other is not, then infer that the recognized object has the higher value with respect to the criterion" (Goldstein & Gigerenzer, 2002, p. 76). *Criterion* refers to a characteristic of reality about which one must judge or decide (e.g., population). In this example, Hamburg would be chosen more frequently because it tends to be more generally recognized, and since this city is indeed the most populated, this would yield average success rates above random chance. The recognition heuristic predicted the results of the 2003 and 2005 Wimbledon tennis matches with equal or greater precision than experts and ATP rankings (Scheibehenne & Brödder, 2007; Serwe & Frings, 2006); in the North American and German stock markets, the heuristic’s average performance equaled or surpassed that of major investment funds, market indices, random stock portfolios and portfolios built with stock from less recognized companies (Borges, Goldstein, Ortmann, & Gigerenzer, 1999). Recognition-based judgments applied to predicting electoral results of political parties have performed as well as or better than opinion polling (Gaissmaier & Marewski, 2011). When both of the elements involved in a decision or all of the elements involved in a judgment can be recognized, the recognition heuristic is no longer applicable, forcing subjects to either search their memories for additional information or change the heuristic (for an introduction to various strategies, see Pohl, 2011). Published studies postulate that when no element is recognized, choosing or judging at random are the only remaining options (e.g., Gigerenzer & Goldstein, 1996; Goldstein & Gigerenzer, 2002; Pohl, 2011).

However, human beings find themselves routinely compelled by social, political and economic circumstances to make decisions or estimations based on various criteria (e.g., distances, weights, prices, ages, manufacturing qualities, ethnic origins, ethical issues, intentions) that involve new and unrecognized elements (e.g., faces, ideas, consumer goods, physical spaces, surnames). Example scenarios in a social context include picking the right people out of a group of strangers for a given task, evaluating the best way to act in a new place or situation; assessing which new foods, movies, books or music to consume; judging among different possible solutions to new problems; etc.

## Overview of the Present Study

In the present study I re-analyzed part of the data presented in Campitelli and Labollita (2010). Said article reports a study in which participants were asked, among other tasks, to estimate the nationality and Elo^2^ rating of a set of chess players on the sole basis of their surnames. One of the tables contained only non-Argentine players, including players with Russian nationality and non-Russian players with Slavic surnames.^3^ Another table contained only Argentine players, some of them with Slavic surnames. The participants recognized almost none of the players' surnames.

Seeing that the estimation of nationality and Elo rating yielded success rates above chance, Campitelli and Labollita conjectured that the participants must have resorted to indirect knowledge (inferences) for their answers (e.g., they may have assigned *Russia* to an unrecognized Karpov on the basis of the suffix -*ov;* they may have estimated a high Elo rating for him on the assumption that Russians —or those with the suffix -*ov*— are better at chess). The authors assumed that the participants: (a) had found some surnames that “seemed Russian” (p. 187); (b) knew that "surnames ending in -*ov* tend to be Russian" (p. 186); (c) knew that Russians "are good at chess" (p. 184); (d) knew that "Russians have been dominating chess for a long time" (p. 188); (e) had used this knowledge to infer nationalities; (f) had used this knowledge to infer Elo ratings.

The goal of this study was to test those assumptions with the purpose of providing evidence regarding how decisions and judgments are made in the face of unrecognized elements, and the environments that facilitate this process.

### Hypothesis

(1) In the estimation of nationality, the surnames of Slavic origin (surnames ending in -*ov*, -*ev* and -*enko,* listed in Table A.1 in Appendix A) will lead to significantly higher average success rates for Russian players than for non-Russian players; (2) In the estimation of Elo ratings, the surnames of Slavic origin (surnames ending in -*ov*, -*ev*, -*enko* and -*cki*, listed in Table A.2 in Appendix A) will lead to significantly higher average success rates for Slavic players than for non-Slavic players.

To theoretically support these hypotheses, I will now describe: how humans can use inferences and heuristics to decide and estimate on the basis of limited information; why these heuristics are ecologically rational; the probabilistic structure of the environments in which they are developed, particularly the recognition heuristic; the components necessary (and the evidence that supports them) to compose a heuristic capable of yielding success without recognition; the ecological rationality of these heuristics with regard to those environments. Finally, I will offer an explanation as to how all these elements may have facilitated the inference of the Russian players’ nationality and the Slavic players’ relative Elo ratings.

## Inferences and Heuristics with Limited Information

*To infer* is to arrive at a conclusion from another certain element (Real Academia Española [RAE], 2012). For example, looking into someone’s eyes to infer a suspected guilt, or inferring the size of a toad from the loudness of its croak (Gigerenzer & Goldstein, 1996). These cues are unreliable because the eyes can deceive, and a small toad can have a deep croak. *Heuristic* means “to find or discover”. Humans use heuristics to guide their decisions and solutions when facing limitations of time, information and cognitive resources, or simply because of their adaptiveness to a given environment (Gigerenzer & Brighton, 2009; Goldstein & Gigerenzer, 2002). For example, baseball players use the gaze heuristic to estimate where the ball will fall (Gigerenzer, 2007).

## Heuristics and Environmental Structures

**Figure 1:**
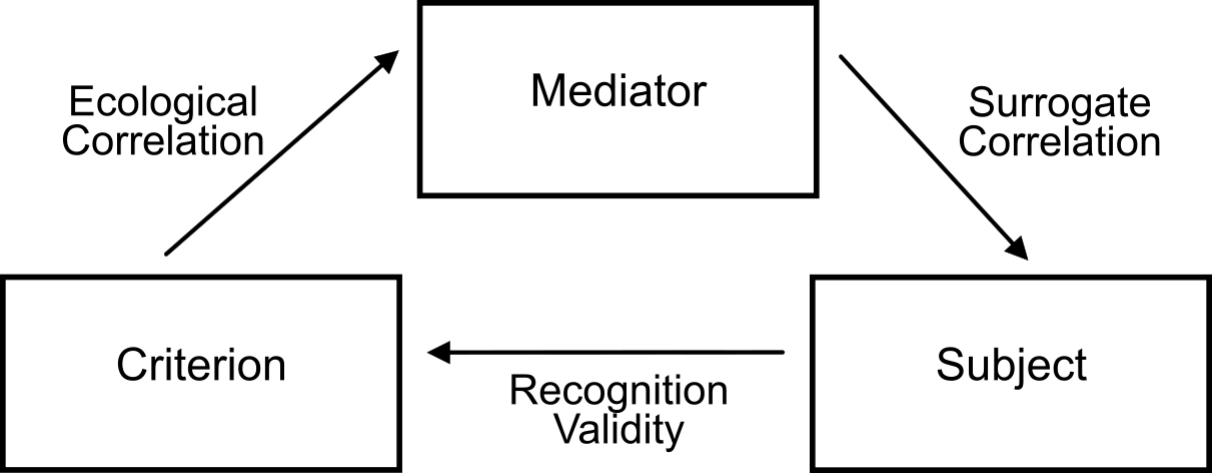
Ecological rationality of the recognition heuristic in a probabilistically structured environment. *Note*. An inaccessible criterion (e.g., population) is reflected by a mediator (e.g., number of mentions in newspapers) that influences recognition probability. The subject can use recognition to infer the criterion.

**Figure 2:**
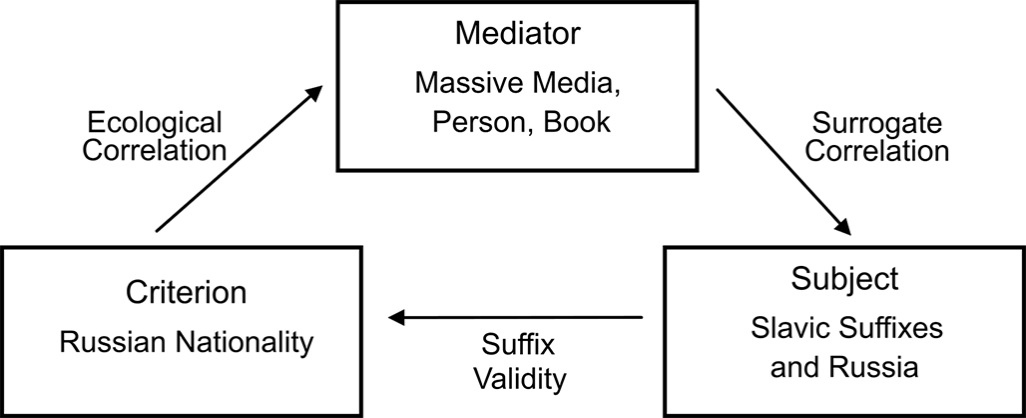
The probabilistic structure of an environment that may enable the inference of the Russian nationality of a surname, without recognition: the inaccessible criterion is reflected by an accessible mediator (e.g., the frequency with which surnames with Slavic suffixes —not only Russian— are reported by the media in proximity or in reference to *Russia*. Thanks to the mediator, the subject associates the suffixes with Russia and uses them later to infer this nationality.

*Ecological rationality*, as it applies to a heuristic, refers to a model of rationality based on correspondence and to the way humans correlate with their environment (Brunswik, 1943; Hammond, 2001; Simon, 1990). For example, the recognition heuristic is based on an ecologically rational cognitive adaptation (Goldstein & Gigerenzer, 2002), since recognition memory is expandable, sensitive and reliable enough to act as a basis for inferences (Galef, 1987; Pfennig, Gamboa, Reeve, Reeve, & Ferguson, 1983; Standing, 1973). However, in any given environment some data will be usually unavailable (e.g., which city has a higher relative population size). Figure 1 (compare with Figure 2-2 of Goldstein & Gigerenzer, 1999, p. 42) models the probabilistic relationship of the elements in an environment appropriate for the recognition heuristic. An inaccessible criterion (e.g., a city’s population) is reflected by a mediator (e.g., newspapers, where usually the most populous cities are mentioned), thus influencing the probability of recognition by the subject (cities mentioned more frequently tend to have higher recognition rates), who will use it later to infer the criterion. A recognition validity of 1 indicates that a recognized city always has more inhabitants than an unrecognized one.

## Heuristics Without Recognition

Although most published studies agree that participants must be able to recognize the element or elements presented to them before they can access additional information, the participants involved in the present study seem to have managed the latter despite the lack of recognition. I posit that the environment they were exposed to before the study of Campitelli and Labollita (2010) must have reflected said inaccessible data through their components and relationships, from which the participants extracted the information they needed to derive their subsequent inferences. I will thus consider: (a) the possibility that surnames in that environment had included cues, more precisely suffix morphemes (hereinafter *suffixes*), linguistic elements located at the end of a word; (b) the capacity of human beings to detect proper names in an environment; (c) the ability to detect and record suffixes in words, including proper names; (d) the ability to detect and record regularities and linguistic relationships, including proper names and morphemes; (e) that certain experiences may have favored the association between Slavic suffixes (such as the Russian -*ov* and -*ev*, but also the Ukranian -*enko*) and Russian nationality above other nationalities; (f) that certain experiences may have favored the association between Slavic suffixes (not only the Russian ones) and chess. Assuming that participants had no prior knowledge of these cognitive and environmental aspects, it can be surmised that they were able to detect and register said aspects without direct instruction and through implicit learning.^4^

Indeed, Slavic surnames have characteristic suffixes (Álvarez, 1968). For example, in Russia it is common to add -*ov* to the name of a child (also -*in* and -*ich*); in Poland, -*sky* or -*skiy*; typical of Ukraine are -*nko* and -*enko*. However, no univocal correspondence can be drawn between suffixes and nationalities, for suffixes can spread geographically. Human beings are able to detect surnames (e.g., Birch & Bloom, 2002; Gelman & Taylor, 1984), as well as their suffixes (e.g., Crepaldi, Rastle, & Davis, 2010) without instruction, unconsciously and after only brief exposures. In these same conditions they can detect linguistic regularities, such as that Slavic suffixes tend to appear around occurrences of *Russia* and related terms, such as *Russian*, *USSR* (e.g., Marcus, Vijayan, Bandi Rao & Vishton, 1999), and create relationships between those elements in their memories (e.g., Pruden, Hirsh-Pasek, Golinkoff, & Hennon, 2006). During the Cold War, the tension between the USSR and the United States enabled a global environment that favored associations between Slavic suffixes and Russian nationality above other Slavic nationalities (Calvert, 2011; Dietrich, 2005; Gerbner, 1991; Mertin, 2008). Finally, two different associations derived from environmental features may have led participants to assign more Elo points to the chess players with Slavic-looking surnames, that is, with the suffixes -*ov*, -*ev*, -*enko* and -*cki* (including players like Gashimov, from Azerbaijan, and the Argentines Kovalyov and Zarnicki), not just the Russian players with surnames ending in -*ov* and -*ev*: (1) The association of Russia with chess, which resulted in the estimation of higher ratings for Russian-looking surnames, with suffixes used as cues for their identification; or (2) The association of certain suffixes with chess, which resulted in the estimation of higher ratings for the surnames that included said suffixes. Let's consider the plausibility of each scenario. The dominance held first by the USSR and later by Russia in the field of international chess (Riordan, 1993) is reflected by the fact that between 1948 and 1991 almost all the chess world champions were Soviet, while from 1992 to 2007 they were mainly Russian and of other Slavic nationalities. A large number of players in the top 50 of the world ranking from 1970 to 1999 were Soviet, and after 1991 from Slavic countries (Howard, 1999). However, the same data supports the alternative scenario: countless surnames with Slavic suffixes were linked to world-class chess performance, some persistently so. The suffix -*ov* was found in the surnames of five of the nine world champions between 1975 and 2006. See the supplementary material for a more in-depth discussion about the topics covered in this section.

## Inference of Russian Nationality

Figure 2 shows the environmental and cognitive characteristics that may have led the participants to infer Russian nationality.

The criterion inaccessible to the subject (the Russian nationality of a player) is reflected by an accessible mediator (the frequency with which the surnames with Slavic suffixes —not only Russian— are reported in proximity or in reference to *Russia*). The subject thus associates the suffixes with Russia, and uses them later to infer this nationality. This is an environment of high uncertainty, as defined by Brunswik (1943), Hammond (2001) and Simon (1990): a given suffix may not always appear clustered near a single nationality, or it could prove insufficient for discriminating between countries, or the frequency of its occurrences in the proximity of *Russia* or related terms might not be representative of its prevalence in Russian surnames. The ecological correlation indicates how frequently surnames with Slavic suffixes are mentioned by the mediator in proximity or in reference to *Russia* (a correlation of 1 means it happens always). The substitute correlation indicates to which extent those mentions match the associations that the subjects created between the suffixes and Russia (in contrast with the recognition heuristic, the frequency of media reports on the specific surnames presented in Campitelli and Labollita, 2010, is nor relevant for this study, as participants were not able to recognize them). The *suffix validity* referred to in Figure 2 indicates the actual relationship between Russian nationality and surnames with Slavic suffixes in that external environment (it could be calculated for one or for the whole set). In this task, the suffix validity shows the extent to which surnames with Slavic suffixes actually belonged to Russian players and thus allowed the inference of their nationality. For example, the suffix -*ov* got .43, as three of the seven foreigners with surnames ending in -*ov* were Russian (Table 5). A heuristic like "all surnames ending in -*ov* are Russian" would yield 43% of correct inferences and 57% of incorrect ones, but it would still outperform a random assignment of Russian nationality. In light of the cognitive and environmental characteristics described, the consistent association of Slavic suffixes (Russian and non-Russian) with Russia is to be expected.

## Inference of Ratings for Slavic Players

**Figure 3:**
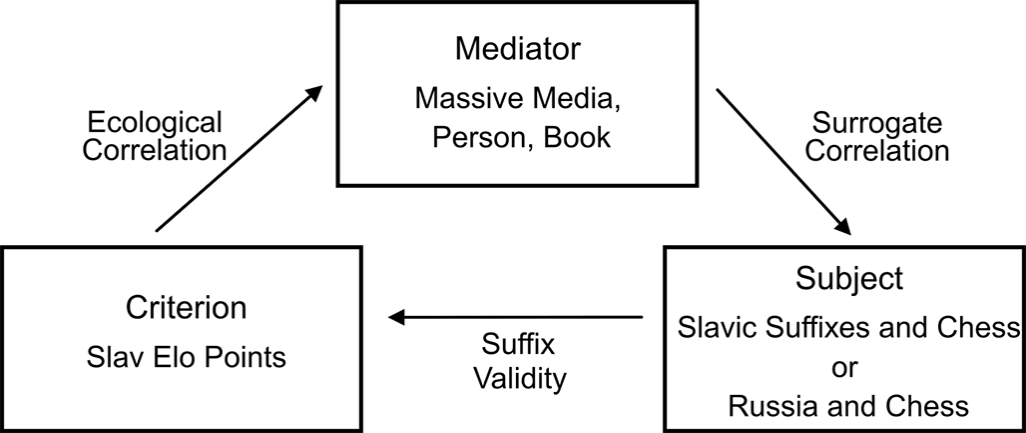
The probabilistic structure of an environment that may result in the inference of higher Elo ratings for Slavic players, with no recognition: the inaccessible criterion is reflected by an accessible mediator (e.g., the frequency with which surnames with Slavic suffixes —not only Russian— are reported by the media in the context of chess, usually in proximity or in reference to *Russia*). Thanks to the mediator, the subject associates Slavic suffixes or Russia with chess, which leads to the inference of more points for chess players whose surnames include a Slavic suffix or who are Russian (using the suffix as the sole basis for discrimination).

Figure 3 shows the environmental and cognitive characteristics that may have led the participants to infer a higher Elo rating for the Slavic players.

The criterion inaccessible to the subject (the Slavs’ higher relative ratings) is reflected by an accessible mediator (the frequency with which surnames with Slavic suffixes —not only Russian— are mentioned by the media in the context of chess, usually in proximity or in reference to *Russia* and related terms). The subject thus associates these suffixes or Russia with chess, which leads to the inference of a higher relative rating for chess players whose surnames include Slavic suffixes or who are Russian (using the suffix as the sole basis for discrimination). There’s uncertainty here as well: it may be that not all players with Slavic suffixes mentioned by the media have relatively higher Elo ratings, or they might be mentioned for reasons other than their performance in chess. The ecological correlation indicates the frequency with which Slavic suffixes are mentioned by the media within the context of chess and in proximity or in relation to *Russia* and related terms; the substitute correlation indicates to what extent these mentions correspond with the subject’s associations. The suffix validity measures the actual relationship between Slavic suffixes and Elo ratings for this external environment. In this task, it represents the proportion of players with Slavic suffixes that actually had a comparatively higher Elo rating. Table 5 shows that the rank correlation between all players with Slavic suffixes and their actual points was .44, which suggests that the heuristic of assigning them comparatively more points generally leads to better estimates.

## Method

In the present study I re-analyzed part of the data presented in Campitelli and Labollita (2010). That article reports a study in which participants were presented with the cognitive reflection test (Frederick, 2005; a number of risky choice and intertemporal decision-making tasks), and a number of judgment tasks. In this article I’ll focus on the task of judging the nationalities and Elo ratings of a group of chess players. I will explain the relevant aspects of Campitelli and Labollita (2010)’s study, and the specific aspects of the current re-analysis.

### Participants

The study by Campitelli and Labollita (2010) included 157 volunteers —47 men and 110 women— from the metropolitan area of Buenos Aires. The average age was 24.4 (*SD* = 5.4, range 16-43). Of the 157 participants, I excluded the 34 that recognized at least one of the chess players, as my interest lies only with those who declared not to recognize any. I then removed the few illegal values received (e.g., estimated Elo ratings below or above the limits set in the instructions — see Instruments section) without disqualifying the participants; outliers were preserved. One participant who submitted too many illegal responses was excluded. The mean age of the remaining 123 participants is 23.8 (*SD* = 4.9, range 16-43).

### Material

The participants of Campitelli and Labollita (2010) were given a booklet they had to fill out with their answers. The section of interest for the present study included four tables (two of them built randomly, two systematically) containing surnames of chess players. Two were filled in with names of Argentine chess players, and the other two with names of non-Argentine chess players. The present analysis considers only the following: (a) the systematic-design table containing non-Argentine players (the only table that included non-Argentine players of Russian nationality along with players with Slav surnames) and (b) the systematic-design table containing Argentine players (the only table that included Argentine players with Slav surnames).

The tables had four columns. The first column contained the names of the players. In the second column, the participants had to indicate whether they recognized each player; in the third column they had to indicate the nationality of each one (nationality information was pre-filled in the table with Argentine players); in the fourth column they were asked to indicate their estimated Elo ratings. Appendix A includes adapted versions of these tables, as well as two extra tables with the correct responses (which, of course, were not accessible by the participants).

### Variables

I coded all the items shown in Table A.1 and Table A.2 of Appendix A in the following variables: (a) Actual Elo rating (*1200* to *2850*), (b) ArgNonArg (*Argentine/non-Argentine*), (c) RusNonRus (*Russian/non-Russian*), (d) SlavNonSlav (*Slavic/non-Slavic*), and created a number of dummy variables for the suffixes: (e) Suffix -ov (*with* -*ov/without ov*), (f) Suffix -ev (*with* e*v/without ev*), (g) Suffix -enko (*with -enko/without -enko*), (h) Suffix -cki (*with -cki/without -cki*). Also, for each player, it was codified (*1*/*0*) the success or failure of the judgment of their nationality. The dependent variables were: (a) nationality accuracy (i.e., whether the participant assigned the nationality correctly), and (b) estimated Elo rating.

For nationality estimation, the mean (*M*) hit rate was calculated for all the values of each independent variable. In the case of the Elo rating, the mean estimated rating was calculated for each value of each independent variable. In both cases, the standard deviation (*SD*) and the standard error (SE) of the mean were calculated for all levels or values, with a confidence interval (CI) of 95%. Correlations were used to analyze the existence, direction and strength of the relationships between the variables. To test the hypotheses, inferential analyses were performed using *t* tests for paired or related samples (the data to be compared came from the same participants), with a significance (α) of .05. The *p* values were obtained through two-tailed tests, and the size of the Cohen effect *d* was calculated.

## Results

### Estimation of Russian Nationality

The 123 participants were able to correctly estimate the nationality of all players in 21.5% of the cases. Table 1 shows the hits disaggregated by variable.

**Table 1:** Nationality Hits, Disaggregated by Variable

Variable	*M*	*SD*	95% CI
RusNonRus			
Russians	0.45	0.35	[0.39, 0.51]
Non-Russians	0.11	0.08	[0.09, 0.12]
Suffix -ev			
With -ev	0.33	0.47	[0.25, 0.42]
Without -ev	0.21	0.13	[0.18, 0.23]
Suffix -enko			
With -enko	0.36	0.48	[0.27, 0.44]
Without -enko	0.21	0.13	[0.18, 0.23]
Suffix -ov			
With -ov	0.23	0.18	[0.20, 0.26]
Without -ov	0.21	0.14	[0.18, 0.23]
Combined			
Russians with -ov	0.52	0.42	[0.45, 0.60]
Russians without -ov	0.35	0.38	[0.28, 0.41]

*Note. N* = 123.

The success rate was significantly higher for the Russian players than for the non-Russians; *t*(122) = 11.26, *p* < .001, Cohen’s *d* = 1.02.

To investigate the extent to which the Slavic suffixes correlated with the correct estimation of Russian nationality, I compared the hits obtained by the one player whose surname contained the suffix -*ev*, corresponding to the Russian Bareev, with the hits of the players without said suffix; that is, everyone else. The differences were significant: *t*(122) = 3.14, *p* < .001, Cohen’s *d* = 0.28^5^.

The success rate of the one surname with the suffix -*enko*, corresponding to the Russian Jakovenko, was significantly higher than the success rates of the surnames without said suffix; *t*(122) = 3.75, *p* < .001, Cohen’s *d* = 0.34.

As expected, the difference in success rates between surnames with the suffix -*ov* and surnames without it was not significant; *t*(122) = 1.35, *p* = .18, Cohen’s *d* = 0.12. Unlike the two surnames with the suffixes -*ev* and -*enko*, not all surnames with the suffix -*ov* belonged to Russian players. Table 2 shows virtually no hits for the surnames ending in -*ov* that belonged to non-Russian players.

**Table 2:** Nationality Hits, Disaggregated by Player

Player	Actual country	*M*
Karpov	Russia	0.55
Malakhov	Russia	0.54
Wang	China	0.50
Tregubov	Russia	0.47
Jakovenko	Russia	0.36
Bareev	Russia	0.33
Gustafsson	Germany	0.29
Adams	England	0.20
Van Wely	Holland	0.13
Ponomariov	Ukraine	0.02
Timman	Holland	0.02
Carlsen	Norway	0.02
Nielsen	Denmark	0.01
Gashimov	Azerbaijan	0.00
Aleksandrov	Belarus	0.00
Topalov	Bulgaria	0.00

I then compared the surnames of Russian players that included the suffix -*ov* with all the surnames that didn’t include it: the difference was significant, *t*(122) = 9.16, *p* < .001, Cohen’s *d* = 0.83; this despite the fact that the set of surnames without *-ov* included surnames with the suffixes -*ev* and -*enko*, both of which had yielded significant success rates when tested against surnames without those suffixes.

To more accurately establish the influence of the suffix -*ov*, I compared all the Russian surnames that included the suffix *-ov* with the Russian surnames that did not, i.e., surnames with the suffixes -*ev* and -*enko*, both of which had yielded significant success rates. The results remained significant: *t*(122) = 4.68, *p* < .001, Cohen’s *d* = 0.42.

Table 3 shows the suffix validity for Russian nationality expressed as proportions and percentages, as well as the success rate for each suffix.

**Table 3:** Suffix Validity for Russia, and Correct Estimation of Russia

Suffixes	Russians/total	Validity (%)	Success rate for Russians (%)
All	5/9	56	45
-*ev*	1/1	100	33
-*enko*	1/1	100	36
-*ov*	3/7	43	52

*Note.* All = *non-Argentines* with surnames ending in -*ev*, -*enko* and -*ov*. *Russians* = number of Russian players with those suffixes. Total = number of players (*Russians* + *non-Russians*) with surnames including those suffixes (1 with -*ev*, 1 with -*enko*, 7 with *-ov;* 9 in total). Validity = percentage of *Russians* with those suffixes against the total number of players with those suffixes. Success rate for *Russians* = percentage of hits for Russian players with those suffixes.

The second and third columns show the actual number of players of Russian nationality in each suffix group; the fourth column shows the percentage of players identified as Russian by the participants. Thus, the validity of Russia for the whole set of suffixes was 56%, since five of the nine surnames with Slavic suffixes belonged to Russian players. The participants correctly assigned 45% of that 56% to Russia. These successes reveal the strength of the associations binding these suffixes to Russia in the participants’ memories, which influenced the level of adherence to the applied heuristics (e.g., when presented with the suffixes -*ev* and -*ov*, only 33% of the participants applied a heuristic such as “*ev* is a Russian suffix,” whereas a heuristic like “*ov* is a Russian suffix” was much more prevalent: the set of Russian players with surnames ending in -*ov* yielded a success rate of 52%).

### Estimation of Elo Ratings for the Slavic Players

Averages for both estimated and actual Elo ratings were calculated for all chess players, showing a correlation *r*(123) = .70 between these variables. Table 4 shows the analysis of the estimated ratings.

**Table 4:** Estimated Elo Ratings, Disaggregated by Variable

Variable	*M*	*SD*	95% CI
All players	2484.94	106.38	[2465.98, 2503.95]
SlavNonSlav			
Slavs	2509.72	114.87	[2489.22, 2530.23]
Non-Slavs	2466.71	111.64	[2446.78, 2486.64]
ArgNonArg			
Argentines	2435.52	136.44	[2411.17, 2459.87]
Non-Argentines	2516.20	121.63	[2494.49, 2537.91]
Suffix -ov			
With -ov	2519.93	122.18	[2498.12, 2541.73]
Without -ov	2469.41	109.74	[2449.83, 2489.00]
Combined			
Non-Argentines with -ov	2530.03	130.21	[2506.79, 2553.27]
Non-Argentines without -ov	2505.57	130.73	[2482.23, 2528.90]
Non-Argentines non-Slavs	2507.89	133.23	[2484.11, 2531.67]
Argentines Slavs	2451.96	171.96	[2421.27, 2482.66]
Argentines non-Slavs	2430.85	137.14	[2406.37, 2455.33]
Argentines with -ov	2451.03	178.64	[2418.73, 2483.32]
Argentines without -ov	2443.84	146.55	[2417.34, 2470.33]

*Note. N* = 123.

The participants tended to assign more points to the Slavic players. A *t*-test on paired samples of estimated ratings for the Slavs and for the non-Slavs showed significantly higher estimates for the former: *t*(122) = 2.89, *p* < .001, Cohen’s *d* = 0.56.

At least part of this difference could be explained by the low ratings estimated for Argentines overall, considering that the set of Slavic players included only two Argentines (Kovalyov and Zarnicki) against the eight Argentines included with the non-Slavs. I then compared the average estimated ratings for Argentines and non-Argentines, which indeed revealed that rating estimations for non-Argentines had been significantly higher: *t*(122) = 6.15, *p* < .001, Cohen’s *d* = 0.56.

To ascertain the extent to which Slavic and Argentine origins accounted for the difference seen in the ratings estimated for Slavs and non-Slavs, I investigated the relation between suffixes and the ratings estimated for the Slavic players. The surnames with the suffix -*ov* had got significantly higher estimates than those without the suffix *ov;* i.e., all the other surnames: *t*(122) = 6.46, *p* < .001, Cohen’s *d* = 0.58.

Because again there were two Argentines among the players with surnames ending in -*ov* and eight Argentines among the rest, it was clearly feasible that the lower ratings estimated for the Argentines in general had brought down the relative ratings of the group without the suffix *-ov*. However, even the ratings estimated for the non-Argentines with surnames ending in -*ov* were significantly higher than the ratings estimated for the non-Argentines with surnames not ending in -*ov*; *t*(122) = 2.89, *p* = .005, Cohen’s *d* = 0.26.

By contrast, the surnames with the suffixes -*ev* and -*enko* received the lowest estimates among the non-Argentine Slavs, and ranked in the bottom four of all non-Argentine players. To find out if these ratings accounted for the significantly lower estimates obtained by the surnames of non-Argentines that didn’t include the suffix -*ov* compared to those non-Argentines that did, I compared the surnames of non-Argentines ending in -*ov* against the surnames of the non-Argentines that didn’t have a Slavic origin (i.e., excluding Bareev and Jakovenko): even here, the non-Argentines with surnames ending in -*ov* showed higher ratings than the rest of the non-Argentines; *t*(122) = 2.57, *p* = .011, Cohen’s *d* = 0.23.

The Slavic Argentines, however, did not get higher ratings than the non-Slavic Argentines; *t*(122) = 1.93, *p* = .056, Cohen’s *d* = 0.17.

Despite the relation between the suffix -*ov* and higher estimated ratings, the Argentine player whose surname included the suffix -*ov*, Kovalyov, did not get a higher rating than the rest of the Argentines; *t*(119) = 0.61, *p* = .545, Cohen’s *d* = 0.06.

Table 5 correlates suffix validities with actual Elo ratings for this task, and shows the relationship between suffixes and estimated Elo ratings.

**Table 5:** Suffix Validity Against Actual Elo, and Relation Between Suffixes and Estimated Elo

Suffixes	Validity Against Actual Elo	Relation with Estimated Elo
All	.44	.43
*-ov*	.41	.47

*Note.* All = -*ev*, -*enko* and -*ov*. Numbers express correlations.

The second column shows the associative strength of the Slavic suffixes with the actual ratings of the players whose surnames contained those suffixes, and summarizes their relative position as shown in Table B.1 in the Appendix B. The rank correlation of .44 shows that the Slavic players chosen by Campitelli and Labollita (2010) tended to have higher real-world Elo ratings than the non-Slavs. The correlation of .43 shown on the third column illustrates the relational strength of these elements in the participants' memories. The actual Elo ratings of the players with surnames ending in -*ov* was generally higher than the ratings of the rest of the players, and the participants thought along the same lines: the correlation between the estimated and actual ratings of the players with surnames ending in -*ov* was .47 (.61 between estimated and actual ratings for Slavic players; .70 between estimated and actual ratings for all players).

## Discussion

### Inference of Russian Nationality

Even without recognizing the players’ names, the participants correctly estimated Russian nationality to a significantly higher degree than other nationalities. I propose that the participants resorted to previously created memories that allowed them to detect the suffixes -*ov*, -*ev* and -*enko* present in the surnames, and evaluate them as useful based on basic cognitive abilities and environmental characteristics, namely: the suffixes -*ov*, -*ev* and -*enko* appear in Slavic surnames; the participants were able to identify surnames without direct instruction and without intention; they could also indirectly and implicitly identify and retain regularities appearing in surnames, such as suffixes, after only a handful of brief exposures (Marquis & Shi, 2012; Pacton, Fayol, & Perruchet, 2005). The heuristics based on the three Slavic suffixes (not only the Russian -*ov* and -*ev*) may have resulted in the higher success rates seen in the estimation of Russian nationality, even though the suffix -*ev* showed the weakest relation, followed by the suffix -*enko* (whose link to Russia was corroborated despite being Ukrainian). The Russian players whose surnames included the suffix -*ov* scored a higher number of hits than the rest of the players (including Russians with surnames ending in -*ev* and -*enko*), corroborating that -*ov* was not associated to nations such as Gashimov’s Azerbaijan or Aleksandrov’s Belarus.

#### Alternative Explanations

Considering that participants were asked to estimate the nationality of chess players, it is plausible to think that mass-assigning Russian nationality to all players based on the association between Russia and chess would have sufficed to get success rates above random chance. However, the evidence suggests this was not the strategy applied, given that:

- The hit rate for the Russian players would have been 100%, and not 45%.
- The hit rate for the non-Russian players would have been 0% and not 11%.
- A quick examination of individual results reveals that no subject estimated the nationality of all Russians correctly without estimating some other nationality correctly as well.

There is another improbable, but not impossible explanation: that the significant success rates were the result of a set of participants massively assigning *Russia* except for some other nationality which they also assigned correctly, while most of the hits for non-Russian nationalities came from another set of participants who did not mass-assign *Russia* and failed to identify any Russian player. This scenario, however, doesn’t account for the significant differences in success rates between the Russians with surnames ending in -*ov* (52%) and the Russians with surnames not ending in -*ov*, i.e., with the suffix -*ev* or -*enko* (35%).

There is another, more unlikely scenario: that a set of participants mass-assigned the Russian nationality except for some other nationality which they also assigned correctly, while the hits for non-Russians were scored by another set of participants who also identified correctly some of the Russian players based on the suffixes in their surnames, particularly -*ov*. This would explain the hit rates for the Russians overall as well as the differences between the Russians with surnames containing the suffix -*ov* and those without it. But this scenario can also be ruled out: even if a second set of participants had acted as described, a quick glance at the results reveals that only 17 subjects correctly assigned the Russian nationality to all Russians. Considering that they also assigned correctly at least one other nationality, it follows that not even them assigned *Russia* blindly.

Could the Russian successes be ascribed to chance? In fact, why did I compare the hits for the Russians with those for the rest of the players, instead of comparing them against random chance? The comparison with chance requires establishing the absolute number of nationalities out of which *Russia* could have been chosen. If all world countries were considered, the probability of choosing *Russia* by chance would be close to 1/200. If we then consider the probability of correctly assigning *Russia* to one or more Russian players by chance, the expected hits would be much lower than those achieved. However, it would be incorrect to assume that the participants knew all the countries in the world. What would be an appropriate number? One hundred nationalities? Fifty? It’s anyone’s guess. A relative comparison with the hits obtained for the other nationalities avoids this problem. If, nevertheless, an approximation is wanted, it could be assumed conservatively that this sample of subjects knew on average 25 nationalities, in which case the probability of choosing *Russia* by chance would be 1/25. Compounding this with the probability of correctly assigning Russian nationality to one or more Russians, the expected successes should still be much lower than those obtained. On the other hand, the request to estimate the nationality of *chess players* could have triggered an association with Russia that eliminated the need for choice, but even the expected success rate of one Russian correctly identified in five attempts would still be significantly lower than the results obtained.

### Inference of Points for the Slavic Players

Even with no recognition, participants assigned significantly higher ratings to the Slavic players with surnames ending in -*ov* than they did to the rest of the players, a result supported by the cognitive abilities and environmental characteristics explained. The Slavs with surnames ending in -*ev* and -*enko*, on the other hand, were assigned comparatively low ratings. The superior performance associated with the suffix -*ov* persisted even when Argentines were excluded from both subsets (they obtained a significantly lower average rating than non-Argentines), and even when the Slavs with surnames ending in -*ev* or -*enko* were excluded from the non-Argentines subset.

#### Alternative Explanations

The reason why chess players with surnames ending in -*ov* were assigned higher ratings may be explained by (a) an association between Russia and good chess performance, which led to the assignment of more points to surnames with the suffix -*ov* because they looked Russian (it was one of the suffixes associated with Russia) or (b) an association between -*ov* and good chess performance, which led to the assignment of more points to surnames with that suffix. The first association is based on Russia's dominance in the field of international chess (Howard, 1999; Riordan, 1993), a fact that has been widely disseminated (Calvert, 2011; Gerbner, 1991). The second association is derived from the historical media coverage of countless players with surnames ending in -*ov*, including world champions such as the very well-known Kasparov. The results support the latter scenario; if the heuristic that produced higher ratings for the surnames with the suffix -*ov* had been based on the connection between Russia and good chess performance, the surnames with the suffixes -*ev* and -*enko* should also have received significantly higher scores in relative terms, since they were correctly identified as Russian. Therefore, the heuristic that in this study led to the estimation of higher average Elo ratings for the Slavs emerged from the association of the suffix -*ov* with good chess performance, an association which is compatible with the human ability to detect and retain relationships between elements of different words based on repetition and proximity, implicitly, and without direct instruction.

#### Compensatory Usage

The ratings estimated for the Argentines were significantly lower than for non-Argentines, a result which reflects Argentina’s middling performance in the field of international chess and the relatively low Elo ratings achieved by Argentines compared with other nationalities (see Appendix B, Table B.1). Not even the single Argentine player that did have a surname ending in -*ov* (Kovalyov) scored significantly higher points than the rest of the Argentines. Although the evidence is limited to a sample of a single player, the heuristic that led to the assignment of high ratings to the surnames with the suffix -*ov* may have been applied to Kovalyov in a *compensatory* way (as opposed to *non-compensatory* or indiscriminate — Kurz-Milcke & Gigerenzer, 2007). The temporary suspension of a heuristic on account of additional knowledge (the Argentine nationality as pertains to chess performance) and the compensatory use of additional cues for judgment-making is consistent with previous studies (e.g., Pachur & Hertwig, 2006; see Pachur, 2011, for a broad discussion).

### Detection and Processing of Suffixes

It may be tempting to attribute to recognition the participant’s detection, and at least part of their processing, of the suffixes present in the surnames of the chess players. It is advisable, however, not to yield to this temptation: not only is the data collected in this study insufficient, but also the disagreements in the current theoretical field would make it difficult to attribute their detection and processing to familiarity, fluency, availability, recognition, etc. (see the supplementary material for a more in-depth discussion).

However, these existing controversies make the findings in this research even more relevant, since the use of linguistic cues in the large number of heuristics studies where the participants *recognized* the elements presented cannot be ruled out. The implications are huge, since at least part of these investigations might have included variables that introduced confusion or interactions unsuspected until now. For example, many city names used in recognition tasks may have contained suffixes (perhaps also prefixes or infixes) that influenced the participants’ decisions when facing not only two unrecognized cities, but also two recognized cities or even a recognized city and an unrecognized one. Additionally, in each case these morphemes might have exerted some influence before or after the subjects detected the degree of familiarity, fluency, availability, recognition, etc., of the cities presented to them.

### Exploitation of the Environment and Ecological Rationality

The results provided evidence on the exploitation of information present in a probabilistic environment external to the laboratory (Brunswik, 1943; Hammond, 2001; Simon, 1990) by way of heuristics that without recognition were ecologically rational thanks to their adaptation to said environment (Gigerenzer & Todd, 1999).

The evidence provided by the successful estimates of Russian nationality is consistent with the environment depicted in Figure 2. Although the value of its ecological correlation could not be obtained, the assumption is that surnames containing Slavic suffixes had been mentioned repeatedly by mediators in proximity or in reference to *Russia* and related terms, with a higher frequency than other nationalities. Although the substitute correlation could not be obtained either, the assumption is that the mediators had disseminated a larger number of surnames with Slavic suffixes in connection with *Russia* and related terms than they did in connection with other countries, although perhaps unevenly: the associative force was greater for the suffix -*ov* than for -*ev* or -*enko*. Table 3 shows the suffix validities for Russia in this task, although the actual validity of each suffix with respect to Russia in that external environment remains unknown (i.e., to what extent people of the world with surnames containing the suffixes -*ov*, -*ev* or -*enko* are actually Russian).

The evidence provided by the assignment of higher ratings to Slavic players in the absence of recognition is consistent with the environment depicted in Figure 3. Although the value of its ecological correlation could not be obtained, it must be assumed that mediators had repeatedly mentioned surnames including the suffix -*ov* in the context of chess (the results do not directly require that these mentions be made in the proximity of or connected with *Russia* and related terms). The value of the substitute correlation could not be obtained either, but it must be assumed that mediators had disseminated at least the suffix -*ov* in the context of chess, since the surnames with this suffix scored significantly higher ratings. Table 5 shows the suffix validities as related to actual Elo points for this task, although the actual validity for each suffix in terms of Elo points is unknown for that external environment (i.e., to what extent chess players of the world whose surnames contain the suffixes -*ov*, -*ev* or -*enko* actually have higher relative ratings).

### Limitations

The re-analysis of the judgment-making task described in Campitelli and Labollita (2010), which required participants to estimate the nationality of chess players, was based on the correct and incorrect estimations of the players’ actual nationalities. Naturally, the answer set included the countries assigned incorrectly as well as the correct ones. Unfortunately, the actual contents of each answer were lost during the digitization process undertaken by the authors, and only their correctness values were preserved. This missing information would have allowed me to analyze in greater depth the link between suffixes and countries, as well as the structure of the environments that generated those associations. For example, while the significant assignment of *Russia* to the Russian player Jakovenko (whose surname contains a Ukrainian suffix) corroborated the hypothesis that the suffix -*enko* would be associated with Russia, we can only guess which countries were picked by the participants with incorrect answers. Ukraine? Which other nationalities were assigned to the Russian players with surnames containing the suffix -*ov*? Were Gashimov, Alexandrov and Topalov, who were not Russian and did not score any hit, also considered Russians? We have no answer for these questions.

The stimuli did not include non-Argentine players with the suffix *cki*, who could have provided additional information about this suffix, nor were included Argentines with the suffixes -*ev* and -*enko*.

No data could be obtained from the real environments in terms of ecological correlation, the substitute correlation, the suffix validity for Russian nationality and the suffix validity for Elo ratings. These would have allowed me to analyze the results in more depth.

It was not possible to carry out an individual, per-subject analysis (it would have required many more answers), which is a mandatory requirement for the study of heuristics according to some researchers of the fast-and-frugal heuristics program (despite the fact that the heuristics presented here were not classified as fast or frugal). For an introduction to the aforementioned requirements, the debate they elicited and the analytical methodology adopted, see Brighton and Gigerenzer (2011); Gigerenzer and Brighton (2009); Gigerenzer and Goldstein (1996); Goldstein and Gigerenzer (2002); Hilbig and Richter (2011).

### Conclusions

According to published studies in the field, random choice and random estimation are the only options for tackling judgment and decision-making tasks where the elements required to infer a criteria are not recognized (e.g., Gigerenzer & Goldstein, 1996; Goldstein & Gigerenzer, 2002; Pohl, 2011). The present re-analysis revealed heuristics that led to the correct inference of Russian nationality and relative Elo ratings for unrecognized chess players. These heuristics were ecologically rational due to the correspondence of their results with reality (Gigerenzer & Todd, 1999) and the relationship of the participants with the environment that facilitated them (Brunswik, 1943; Hammond, 2001; Simon, 1990).

The results support the use of models of limited rationality to characterize human psychology, since participants applied heuristics to achieve success rates above chance level, despite the risk of overestimating the heuristics’ effectiveness and underestimating the participants’ own biases, a behavior justified in evolutionary terms (e.g., Haselton & Nettle, 2006). I thus invite a revision of what is considered “good reasoning” when applied to human beings facing problems in environments of uncertainty that typically call for satisfactory, rather than optimal, solutions.

My hope is that this study will provide a basis for new studies in the field of heuristics under previously unexplored conditions, and a new perspective for the analysis of previous works, towards a better understanding of the relationship between cognition and the environment.

## Appendix A

Instructions and Tables Presented to Participants of Campitelli and Labollita (2010)

In the following two lists you will find names of Argentine and non-Argentine chess players. You must complete the following for each of them:

1. Indicate if you knew a chess player with that name before this investigation.
2. Indicate country of origin for each non-Argentine player.
3. Assign Elo points for each chess player. Keep in mind that chess ranking is measured in points ranging from 1,200 (low level amateurs) to 2,850 (best player in the world).

**Table A.1:** Non-Argentine Players.

Surname	¿Do you know her? (Yes/No)	Country	Elo points
Van Welly			
Nielsen			
Bareev			
Gustafsson			
Jakovenko			
Wang			
Karpov			
Malakhov			
Gashimov			
Aleksandrov			
Tregubov			
Topalov			
Carlsen			
Adams			
Ponomariov			
Timman			

**Table A.2:** Argentine Players

Surname	¿Do you know her? (Yes/No)	Country	Elo points
Villanueva		Argentina	
Flores		Argentina	
Kovalyov		Argentina	
Hase		Argentina	
Lorenzini		Argentina	
Adla		Argentina	
Amura		Argentina	
Zarnicki		Argentina	
Peralta		Argentina	
Luconi		Argentina	

## Appendix B

Chess Players’ Countries and Real Elo Points Presented in Campitelli and Labollita (2010)

**Table B.1:** Countries and Actual Elo Points (Ordered by Elo)

Surname	Actual country	Actual Elo points
Topalov	Bulgaria	2,813
Carlsen	Norway	2,772
Jakovenko	Russia	2,742
Ponomariov	Ukraine	2,741
Gashimov	Azerbaijan	2,740
Wang	China	2,736
Malakhov	Russia	2,715
Nielsen	Denmark	2,687
Adams	England	2,682
Van Welly	Holland	2,651
Tregubov	Russia	2,649
Aleksandrov	Belarus	2,639
Bareev	Russia	2,633
Gustafsson	Germany	2,622
Karpov	Russia	2,619
Flores	Argentine	2,607
Timman	Holland	2,588
Kovalyov	Argentine	2,577
Peralta	Argentine	2,565
Zarnicki	Argentine	2,515
Adla	Argentine	2,498
Lorenzini	Argentine	2,458
Amura	Argentine	2,335
Villanueva	Argentine	2,326
Luconi	Argentine	2,293
Hase	Argentine	2,279
